# Advancing the Frontier: Neuroimaging Techniques in the Early Detection and Management of Neurodegenerative Diseases

**DOI:** 10.7759/cureus.61335

**Published:** 2024-05-29

**Authors:** Ahmed S Akram, Han Grezenko, Prem Singh, Muhammad Ahmed, Baran Dilshad Hassan, Vibhavari Hagenahalli Anand, Abdelrahman A Elashry, Faran Nazir, Rehman Khan

**Affiliations:** 1 Psychiatry, Faisalabad Medical University, Faisalabad, PAK; 2 Medicine and Surgery, Guangxi Medical University, Nanning, CHN; 3 Translational Neuroscience, Barrow Neurological Institute, Phoenix, USA; 4 Neurology, Dow University of Health Sciences, Karachi, PAK; 5 Psychiatry and Behavioral Sciences, Dow University of Health Sciences, Karachi, PAK; 6 Medicine, Hawler Medical University, College of Medicine, Erbil, IRQ; 7 Critical Care Medicine, Aster RV Hospital, Bangalore, IND; 8 Mansoura Manchester Medical Program, Mansoura University, Mansoura, EGY; 9 Internal Medicine, Faisalabad Medical University, Faisalabad, PAK; 10 Internal Medicine, Mayo Hospital, Lahore, PAK

**Keywords:** psychiatry, healthcare disparities, artificial intelligence in healthcare, spect, pet imaging, parkinson’s disease, alzheimer’s disease, neurodegenerative diseases, early detection, neuroimaging

## Abstract

Alzheimer's and Parkinson's diseases are among the most prevalent neurodegenerative conditions affecting aging populations globally, presenting significant challenges in early diagnosis and management. This narrative review explores the pivotal role of advanced neuroimaging techniques in detecting and managing these diseases at early stages, potentially slowing their progression through timely interventions. Recent advancements in MRI, such as ultra-high-field systems and functional MRI, have enhanced the sensitivity for detecting subtle structural and functional changes. Additionally, the development of novel amyloid-beta tracers and other emerging modalities like optical imaging and transcranial ultrasonography have improved the diagnostic accuracy and capability of existing methods. This review highlights the clinical applications of these technologies in Alzheimer's and Parkinson's diseases, where they have shown improved diagnostic performance, enabling earlier intervention and better prognostic outcomes. Moreover, the integration of artificial intelligence (AI) and longitudinal research is emerging as a promising enhancement to refine early detection strategies further. However, this review also addresses the technical, ethical, and accessibility challenges in the field, advocating for the more extensive use of advanced imaging technologies to overcome these barriers. Finally, we emphasize the need for a holistic approach that incorporates both neurological and psychiatric perspectives, which is crucial for optimizing patient care and outcomes in the management of neurodegenerative diseases.

## Introduction and background

Neurodegenerative diseases like Alzheimer's and Parkinson's are diseases with a characteristic progressive degeneration of the structure and function of the central nervous system or peripheral nervous system. The diseases are mainly characterized as being pinpointed within groups of the elderly but not specified by any given age. As such, this has led to its notoriety as being the most common form of dementia characterized by the loss of cognitive function. Contrary to the normal process of all these occurrences, motor function in a Parkinson's disease (PD) patient is dramatically affected due to the breakdown in the central nervous system [1].

The early identification of the onset of these neurodegenerative diseases is crucial for effectively managing these ailments. The onset of the diseases at an early stage might be identified to delay not only the progression of the respective diseases but also to soften them by employing timely intervention, which may include pharmacological treatment and lifestyle adjustment. Early diagnosis also allows for future planning and effective symptom management for patients and family members [2,3].

After much time, neuroimaging has become the pillar of proper diagnosis and has subsequently come to dominate the management of many different neurodegenerative diseases. It provides a major source for methods like MRI and PET scans used in the detection of early signs before the presentation of significant neurological changes. These imaging modalities have developed over time to be high resolution and could offer an early chance for detection, helping the physician regarding disease monitoring with respect to decision-making and tracking progression [4,5].

Moreover, the overlap of symptoms between neurodegenerative diseases and psychiatric conditions presents additional diagnostic challenges. Cognitive decline in Alzheimer’s, as well as mood disturbances and motor function changes in Parkinson’s, can mimic or coincide with symptoms of psychiatric disorders such as depression, anxiety, and psychosis. This overlap complicates the differential diagnosis, making it difficult to distinguish between primary psychiatric disorders and neurodegenerative diseases that present with psychiatric symptoms. Neuroimaging plays a pivotal role in this context, as it helps in distinguishing the underlying causes of these symptoms, thereby guiding more accurate diagnoses and tailored treatment plans.

The focus of this narrative review, therefore, seeks to look into the advances made in neuroimaging techniques and how they have continued to play a pivotal role in the early detection of debilitating neurodegenerative diseases such as Alzheimer's and Parkinson's. This review, therefore, places much emphasis on the recent technological improvement and clinical application, bearing in mind the importance of early detection and its potential to significantly alter disease outcomes. As the following overview will show, it focuses on grounding the review within a context more accurately representing what is currently capable or likely for research, development, and investment regarding neuroimaging for neurodegenerative diseases. This is to inspire further work and investment in this needed area.

## Review

Advances in neuroimaging techniques

MRI Innovations

Magnetic resonance imaging has made substantial progress, especially in the areas of high-resolution imaging and functional MRI (fMRI), resulting in a major improvement in the ability to detect neurodegenerative illnesses at an early stage. An important advancement in MRI technology is the creation of ultra-high-field MRI systems, such as the 7 Tesla (T) MRI scanners. These scanners provide exceptional spatial resolution and signal-to-noise ratio, which were previously unattainable [6]. This enhancement enables the precise visualization of smaller brain regions that were previously difficult to image with clarity. As a result, it aids in the early diagnosis of mild neurodegenerative alterations before they develop into more noticeable symptoms [7]. In a recent study conducted by Wardlaw et al. [8], it was shown that 7T MRI scanners have the ability to identify brain microbleeds that have an average diameter of 0.5 mm. This is a substantial improvement compared to traditional 1.5T or 3T MRI scanners, which can only detect microbleeds with a minimum diameter of 2.5 mm.

Moreover, notable progress has been made in the field of functional MRI. Recent advancements have facilitated far more precise and rapid scans, which are essential for capturing the dynamic activity of the brain. These improvements are particularly notable in investigating the involvement of certain brain regions in different cognitive activities and how these regions are affected by neurodegenerative processes. Improved fMRI technologies can now more accurately detect and track functional alterations in the brain that signal the initial phases of disorders such as Alzheimer's and Parkinson's. A study [9] found that resting-state fMRI may accurately identify alterations in functional connectivity within the default mode network in persons with moderate cognitive impairment (MCI), a common precursor to Alzheimer's disease (AD), with an accuracy of 88%.

Furthermore, MRI advancements have shed light on how neurodegeneration and psychiatric symptoms interconnect through changes in specific brain regions such as the hippocampus and prefrontal cortex. These areas are critical not only in neurodegenerative processes but also in psychiatric conditions like depression and bipolar disorder. Alterations in the hippocampus and prefrontal cortex, often detected through advanced MRI techniques, can be indicative of both cognitive decline and mood dysregulation. This dual relevance makes MRI an invaluable tool in predicting or correlating psychiatric symptoms in patients with neurodegenerative diseases, offering insights that could significantly influence both diagnostic and therapeutic approaches [10].

Risacher et al. [11] conducted quantitative research that showed that changes in brain volume and functional connectivity, evaluated by MRI, can be used to predict cognitive deterioration in people with AD. The analysis had an area under the curve (AUC) of 0.92.

Strengths and Limitations of MRI

The main advantage of MRI is its remarkable capacity to provide high-resolution, intricate images of brain regions without ionizing radiation, making it well-suited for repeated exams in clinical research. Nevertheless, MRI has a lower sensitivity for detecting the molecular alterations that take place in the initial phases of neurodegenerative disorders. This limitation can reduce its usefulness in identifying these diseases before observable morphological abnormalities occur [12].

Positron Emission Tomography and Single-Photon Emission Computed Tomography (SPECT)

Significant progress has been made in the field of PET and single-photon emission computed tomography (SPECT), especially in the creation of novel tracers that enhance the accuracy of detecting different neurodegenerative indicators [13]. Tracers have become essential tools in identifying and comprehending the advancement of diseases like Alzheimer's and Parkinson's. They do this by illuminating the buildup of abnormal proteins such as tau and amyloid-beta. For instance, recently developed PET tracers such as flortaucipir (^18F) have demonstrated potential in identifying the buildup of tau proteins, which are important indicators of the advancement of AD. In a study conducted by Ossenkoppele et al. [14], it was found that flortaucipir (^18F) PET had a sensitivity of 92% and a specificity of 92% in distinguishing AD from other neurodegenerative disorders. This performance was superior to that of the traditional [^18F] FDG-PET, which had a sensitivity of 84% and a specificity of 73%. Advancements in SPECT tracers have enhanced the ability to visualize dopamine transporters, which play a critical role in diagnosing PD.

Moreover, PET and SPECT tracers are increasingly targeting neurotransmitter systems such as serotonin and dopamine, which are crucial in both psychiatric and neurodegenerative diseases. The ability to visualize these neurotransmitter systems enhances the utility of PET and SPECT in a dual role. For example, tracers that target serotonin receptors can help assess changes associated with psychiatric conditions like depression and anxiety, which frequently co-occur with neurodegenerative diseases. This dual utility is pivotal in providing a more comprehensive understanding of the neurobiological changes that occur in these disorders, thereby aiding in the development of targeted therapies that address both the neurodegenerative and psychiatric aspects of patient care.

Strengths and Limitations of PET

The primary advantage of PET is its ability to identify molecular-level biochemical alterations, enabling the early detection of neurodegenerative disorders before the manifestation of structural changes. This capacity is crucial for comprehending the initial development of illnesses and assessing proposed therapies' effectiveness. Nevertheless, PET scans necessitate the utilization of radioactive tracers, which might present safety issues, especially when several scans are performed. Additionally, the expensive nature of PET scans can restrict their availability and widespread implementation in ordinary clinical practice [15].

Emerging Techniques

Explorations into non-traditional imaging technologies have recently uncovered new possibilities for neuroimaging. Optical imaging has been utilized to get detailed images of metabolic and molecular activities in the brain, which could potentially enable the early identification of neurodegenerative alterations [16]. A study conducted by Bouchard et al. [17] showed that near-infrared spectroscopy, an optical imaging technique, can accurately detect changes in cerebral blood flow and oxygenation in AD patients. This technique has a spatial resolution of up to 1 mm, enabling the identification of early functional deficits with an accuracy of 87%. In addition, transcranial ultrasonography has demonstrated promise in detecting certain indicators, such as midbrain echogenicity, which is linked to PD. A study conducted by Walter et al. [18] found that transcranial sonography demonstrated a sensitivity of 90.4% and a specificity of 82.8% in identifying midbrain hyperechogenicity, which is a distinct hallmark of PD. Although still being extensively researched, these approaches can potentially enhance the neuroimaging arsenal by providing less intrusive and more easily accessible options for early diagnosis [19].

Clinical applications

Alzheimer's Disease

Neuroimaging technology developments have fundamentally changed the field of AD diagnosis, especially in terms of detecting the disease in its early and pre-symptomatic phases. Magnetic resonance imaging and PET, including tau and amyloid PET tracers, have been crucial [20].

The high-resolution imaging capabilities of MRI techniques enable precise viewing of brain regions and have been crucial in the early identification of structural abnormalities linked to AD. The capacity of MRI to identify small changes in brain volume in regions such as the hippocampus offers important information long before clinical symptoms appear [16]. A three-year longitudinal study by Dickerson et al. [21] found that, compared to only 5% of those without hippocampal atrophy, those with MCI who exhibited hippocampal atrophy on MRI advanced to AD at a rate of 38%.

With amyloid and tau tracers in particular, PET imaging provides important benefits for deciphering the underlying pathophysiology of AD. Standard diagnostic tools, AD differentiation from other dementias, and disease progression monitoring are now amyloid PET tracers. Because they attach to amyloid plaques, one of the main features of AD, these tracers make them visible in live patients. New PET tracers like flortaucipir have improved the ability to identify tau pathology, which is linked to the course of cognitive deterioration [22]. In separating AD from other neurodegenerative diseases, research by Ossenkoppele et al. [14] found that the combination of amyloid and tau PET imaging had a sensitivity of 98% and a specificity of 88%.

Moreover, the accuracy of AD diagnosis has increased with the combined use of tau and amyloid PET imaging, especially when differentiating it from other neurodegenerative diseases. These tracers' specificity in pointing out the unique patterns of tau and amyloid deposition in the brain facilitates a more accurate evaluation of the severity and stage of the disease [23]. Insel et al. [24] conducted a long-term study that revealed those with MCI who had increased PET scan levels of tau and amyloid were more than twice as likely to develop AD in the next three years than those without these biomarkers.

Neuroimaging has also uncovered significant links between neurodegenerative patterns and psychiatric symptoms such as depression and anxiety in Alzheimer’s patients. For instance, changes in areas like the prefrontal cortex and amygdala, identified through neuroimaging, have been correlated with an increased incidence of mood disorders and emotional dysregulation in these patients. This discovery has profound implications for integrated treatment approaches that address both cognitive decline and psychiatric well-being, suggesting that therapeutic strategies should encompass both neurocognitive support and psychiatric care to manage the holistic needs of Alzheimer’s patients effectively. By incorporating these neuroimaging methods into clinical practice, we have not only improved our knowledge of Alzheimer's pathology but also significantly changed the diagnostic process, enabling earlier and more focused therapies. This comprehensive approach offers the potential to halt the course of the disease through timely and multi-faceted treatment actions, ultimately enhancing patient care and quality of life.

Parkinson's Disease 

Early detection and care of PD have been greatly aided by advanced imaging techniques, especially dopamine transporter (DaT) scanning with SPECT. The unique decrease in DaT availability, especially in the striatum, that these scans may identify helps to distinguish PD from other forms of parkinsonism. In separating PD from other parkinsonian illnesses, DaT SPECT exhibited a pooled sensitivity of 98.6% and a specificity of 93.6%, according to a meta-analysis by Vlaar et al. [25]. Functional MRI has also contributed by pointing out changes in blood oxygenation levels associated with the course of the illness. Specifically in the resting state, this technique, known as functional connectivity, shows promise for identifying early PD-related changes in brain activity [26]. Research [27] showed that using changes in functional connectivity within the basal ganglia network, resting-state fMRI could accurately distinguish early-stage PD patients from healthy controls with 91.7% accuracy.

For patient outcomes, a PD diagnosis made early and accurately matters greatly. In a randomized controlled trial by Schuepbach et al. [28], motor function improved by 38% in PD patients who had deep brain stimulation (DBS) within seven years of diagnosis and by 14% in those who received DBS later. Moreover, early DBS treatment, directed by imaging results, has been demonstrated to enhance PD patients' quality of life and motor performance.

Furthermore, neuroimaging has proven invaluable in detecting brain changes associated with neuropsychiatric complications of PD, such as depression, psychosis, and impulse control disorders. Changes in specific brain regions, notably the frontal cortex and limbic structures, observed through advanced imaging modalities like PET and SPECT, are often linked to these symptoms. Understanding these alterations can guide the development of targeted interventions that address both the neurological and psychiatric aspects of the disease. For example, alterations in dopamine pathways visualized by DaT SPECT can correlate with impulse control disorders, informing more personalized therapeutic strategies. This holistic approach to diagnosis and treatment, enabled by neuroimaging, facilitates a more comprehensive management of PD, aiming to improve overall patient well-being and not just motor symptoms.

Differentiating from Other Movement Disorders

Differentiating PD from other movement disorders, such as progressive supranuclear palsy (PSP) and multiple system atrophy (MSA), depends critically on imaging. In this sense, cardiac 123𝐼123I-metaiodobenzylguanidine (MIBG) SPECT is useful; it shows notable decreases in uptake in PD compared to other diseases [20]. Furthermore, 18𝐹18F-fludeoxyglucose (FDG) PET scans offer comprehensive maps of brain metabolism that, by revealing particular patterns of brain activity and metabolism, might distinguish PD from atypical parkinsonian diseases [23].

Rare Neurodegenerative Diseases

Sophisticated neuroimaging has also improved our understanding of and capacity to diagnose less prevalent neurodegenerative disorders. Particular biomarkers linked to uncommon diseases, including Lewy body dementia and corticobasal degeneration, have been identified using advanced MRI, PET, and SPECT methods [26]. Through the depiction of certain degenerative alterations in the brain made possible by these imaging modalities, precise diagnosis and discrimination from more prevalent diseases like Alzheimer's are aided. Finding these distinctive trends aids in early diagnosis, tracking the course of the illness, and evaluating therapy response [29].

Challenges and limitations

Technical Challenges

Our capacity to identify and treat neurodegenerative illnesses early on has been greatly improved by developments in neuroimaging technology. Technical constraints still exist, nevertheless, especially about faster imaging and higher resolution [30]. Higher resolution is essential to see minute anatomical features that might be early warning indicators of conditions like Parkinson's or Alzheimer's before they become more widely apparent. In people with preclinical AD, higher-resolution MRI scans (0.8 mm isotropic voxels) were shown by Bossa et al. [31] to be able to identify volume changes in the hippocampus and entorhinal cortex up to six years sooner than lower-resolution scans (1.2 mm isotropic voxels). Similarly, quicker imaging methods are required to shorten patient stays in scanners, which is particularly crucial for patient comfort and lowering movement artifacts that can lower image quality [32]. Simultaneous multi-slice imaging is one technique that has been demonstrated to shorten functional MRI acquisition times by up to 60%, improving the ability to identify minute changes in functional connectivity in the early stages of AD [33].

The earlier and more precise diagnosis that can greatly influence patient outcomes and disease management depends on these technological obstacles being overcome. A meta-analysis by Cummings et al. [34] on AD found that, compared to untreated persons, early cholinesterase inhibitor treatment in those with mild cognitive impairment decreased the probability of progression to Alzheimer's dementia by 35% over three years. Furthermore, the identification of Alzheimer's patients employing sophisticated imaging methods has made it easier for them to take part in clinical trials for disease-modifying treatments, which, if started early on, may be able to slow or stop the course of the illness [35].

Ethical Challenges

There are profound moral conundrums associated with early neurodegenerative disease diagnosis, particularly concerning the psychological effects on patients and their families. Knowing years in advance that one has a possibly crippling and irreversible illness can lead to significant psychological suffering. This raises ethical questions about the timing and method of revealing such diagnoses, especially when neuroimaging predicts not only neurological decline but also potential psychiatric conditions such as depression or anxiety, which may complicate the patient's and family's coping mechanisms [36].

Furthermore, the high costs of advanced imaging techniques raise concerns about accessibility and equity in the distribution of healthcare resources. There is an ongoing debate on how to ensure fair access to these cutting-edge diagnostic techniques and how to balance the benefits of early diagnosis with the potential psychological toll it imposes on individuals [37]. The psychological impact of knowing one's likely future mental health challenges adds another layer of complexity to these discussions, emphasizing the need for sensitive handling of information and patient support systems.

Accessibility disparities in neuroimaging technology availability vary greatly across the world, resulting from socioeconomic differences that limit early diagnosis opportunities for neurodegenerative disorders. While developed countries typically have better access to such technologies, low- and middle-income countries struggle due to inadequate resources and healthcare facilities [38]. The World Health Organization (WHO) reports that, while 89% of high-income nations have access to at least one MRI unit, only 14% of low-income countries do [39]. Those from lower economic backgrounds in all countries are less likely to have access to these sophisticated diagnostic tools, exacerbating health outcomes through delayed diagnosis and treatment. This disparity not only delays treatment for neurodegenerative conditions but also for associated psychiatric symptoms, which are less likely to be recognized and managed effectively in these populations [40].

A systematic review by Russ et al. [41] suggested that possibly as a result of delayed diagnosis and treatment, people from lower socioeconomic groups had a 1.6 times higher risk of developing AD and a 1.9 times higher risk of vascular dementia than those from higher socioeconomic groups. This indicates a critical need to address disparities not just in neurodegenerative disease management but also in the associated psychiatric care.

Technical challenges also contribute to these disparities. Early diagnosis of subtle neurodegenerative changes often relies on high-resolution imaging, which requires expensive and complex equipment that is unavailable in many parts of the world. Moreover, ethical dilemmas concerning the benefits versus potential disadvantages of early disease identification are compounded by the psychological effects of such early diagnoses, particularly in contexts where therapeutic options are limited [30]. Addressing these ethical challenges involves improving healthcare infrastructure, ensuring that ethical issues in early detection are addressed, and increasing global access to advanced diagnostic tools [42]. This must include careful consideration of the stigma and treatment implications related to both neurodegenerative and psychiatric diagnoses, ensuring that all patients receive comprehensive and compassionate care tailored to their full spectrum of health needs.

Future directions

Research Trends

Artificial intelligence (AI) and its integration with various imaging technologies have significantly advanced neuroimaging for neurodegenerative illnesses in recent years. The use of AI has notably improved the accuracy and effectiveness of early detection techniques. By analyzing complex imaging data, this technology enables earlier and more precise diagnosis of diseases like PD and AD [42].

Current research efforts are focused on developing AI-driven algorithms capable of detecting subtle imaging patterns that indicate early neurodegenerative changes before they become overt. These applications of AI in analyzing imaging data and health records are being explored for their potential to predict the onset and progression of neurodegenerative diseases much earlier than previously possible [43].

An additional burgeoning area is the combination of AI with biosensing technology. This approach employs wearable and non-wearable sensor devices to monitor physiological activities and behaviors, including sleep cycles, cognitive changes, and motor activity. The continuous, real-time data provided by these devices are analyzed by AI algorithms to identify early warning signs of disease, offering a more dynamic and accurate method to track disease progression [44].

Advanced biosensing systems and brain-computer interfaces are also subjects of ongoing research. These systems are designed to interact directly with brain activity, providing yet another layer of data for AI analysis, which can significantly enhance diagnostic and prognostic capabilities for neurodegenerative diseases [45].

In light of these technological advancements, there is a compelling case for future research directions that involve collaborative efforts between neurology and psychiatry. Such collaborations are crucial to developing comprehensive neuroimaging strategies that not only detect neurodegenerative changes but also consider psychiatric conditions that often co-occur with these diseases. Integrating AI to analyze diverse data types (from neuroimaging and genetic markers to psychiatric assessments) significantly improves the early diagnosis and personalization of treatment plans. This integrated approach promises to enhance our understanding of the complex interplay between neurological and psychiatric symptoms, ultimately leading to more effective and tailored interventions for patients suffering from neurodegenerative diseases [46].

By combining AI with these state-of-the-art technologies, neuroimaging is expected to become even more powerful, completely transforming the early detection and treatment paradigms for neurodegenerative diseases. This integrated research strategy underscores the importance of a holistic view in the management of these complex diseases, where neurology and psychiatry converge to improve patient outcomes.

Clinical Trials and Longitudinal Studies

The need for long-term research and clinical trials that use cutting-edge neuroimaging methods to comprehend the course of neurodegenerative disorders cannot be emphasized enough. These investigations depend on the identification of early biomarkers and knowledge of the natural history of disorders such as Parkinson's and Alzheimer's [35].

Longitudinal investigations with PET imaging have become essential for AD. Amyloid-beta PET tracers are used in these studies; they are essential for monitoring, diagnosis, and grouping disease progression. Comparably, tau-specific PET tracers have created new pathways to comprehend tau pathology in AD, offering information about its dissemination and utility as a secondary outcome measure in clinical studies [38].

One longitudinal observational clinical trial with a focus on PD is the Parkinson's Disease Progression Neuroimaging Initiative (PDPNI). This effort emphasizes the need for routine, comprehensive clinical and imaging assessments to monitor the course of PD and find possible indicators of progression. These studies often combine several evaluations, such as neuroimaging, cognitive testing, and motor function, to provide a thorough picture of the course of the disease [40].

The accuracy and productivity of neuroimaging analysis should be greatly improved by developments in machine learning and AI. According to research by Wen et al. [47], integrating deep learning algorithms could increase the accuracy of AD diagnosis from MRI scans by up to 15% over conventional analytic techniques. Comparably, a study by Zhou et al. [48] projected that the efficiency of Alzheimer's diagnosis and monitoring would greatly increase with the 80% reduction in time needed for amyloid load measurement using AI-assisted analysis of PET images.

Advanced computer models and simulations should also yield important new information about the course of neurodegenerative disorders. Congdon et al. [49] demonstrated the value of early diagnosis and treatment made possible by sophisticated neuroimaging techniques by predicting, using a computational model of tau protein propagation, that early intervention with tau-targeted therapies may possibly postpone the onset of AD by up to 10 years.

Policy and Practice Changes

Future health policy and clinical practice are probably going to be greatly impacted by neuroimaging developments. Diagnostic criteria and treatment paradigms may change as imaging technology advances. For instance, early diagnosis of neurodegenerative disorders should change treatment plans to focus on early intervention and prevention, maybe even before clinical symptoms appear [50].

Public health approaches to aging and cognitive health may need to change to use these technologies in routine screenings and therapeutic settings. Moreover, early diagnosis's social, legal, and ethical ramifications must be considered to guarantee that laws promote both the best interests of patients and technical developments. Researchers, physicians, legislators, and the public must work together to ensure that the advantages of sophisticated neuroimaging are realized and fairly shared, ultimately improving patient care and outcomes in neurodegenerative disorders [51,52].

## Conclusions

The significance of advanced neuroimaging in facilitating the early diagnosis and treatment of neurodegenerative diseases such as Parkinson's and Alzheimer's is underscored throughout this narrative review. Innovations in MRI, PET, and other emerging imaging modalities have notably improved our ability to detect subtle changes in the brain. This enhanced detection capability aids in the diagnosis and enables earlier interventions that may slow the progression of these debilitating diseases. However, the implementation of these technologies faces challenges, including access disparities, ethical concerns, and technical limitations. The integration of AI with longitudinal research also shows great promise in refining early detection techniques.

Addressing these issues is crucial for maximizing the potential of neuroimaging advancements to bring about a shift toward proactive prevention and early intervention in neurodegenerative disorders. Moreover, incorporating a holistic approach that includes psychiatric perspectives is vital. This integrated approach is not merely an enhancement of diagnostic precision; it is imperative for a comprehensive evaluation of the patient. By recognizing and addressing the full spectrum of symptoms, including both neurological and psychiatric, we can improve overall patient outcomes and quality of life. Thus, as we continue to refine neuroimaging techniques and address associated challenges, we must also ensure that these advances are used in a manner that considers the entire well-being of the patient, heralding a paradigm shift towards more personalized and effective healthcare strategies for those suffering from neurodegenerative diseases.
